# Anatomical analysis of the muscle layers of the soft palate in velopharyngeal closure: A cadaveric study

**DOI:** 10.1016/j.jpra.2025.12.025

**Published:** 2026-01-03

**Authors:** Keiko Fukino, Masahiro Tsutsumi, Yuri Kinoshita, Yoshiro Matsumoto, Takashi Ono, Joe Iwanaga, Keiichi Akita

**Affiliations:** a Department of Oral and Maxillofacial Anatomy, Graduate School of Medical and Dental Sciences, Institute of Science Tokyo, Tokyo, Japan; b Morinomiya University of Medical Sciences, Inclusive Medical Sciences Research Institute, Osaka, Japan; c Department of Clinical Anatomy, Graduate School of Medical and Dental Sciences, Institute of Science Tokyo, Tokyo, Japan; d Department of Orthodontic Science, Graduate School of Medical and Dental Sciences, Institute of Science Tokyo, Tokyo, Japan; e Department of Neurosurgery, Tulane Center for Clinical Neurosciences, Tulane University School of Medicine, New Orleans, LA, USA

**Keywords:** Anatomical analysis, Soft palate, Velopharyngeal closure, Palatopharyngeus, Superior constrictor of the pharynx

## Abstract

**Background:**

In general, the levator veli palatini is essential for velopharyngeal closure as it elevates the velum. However, reconstructing the levator veli palatini alone does not fully restore velopharyngeal closure, suggesting that surrounding muscles—particularly the palatopharyngeus and superior constrictor—are also essential. We aimed to clarify the spatial relationships between the muscle bundles of the soft palate and pharynx macroscopically and histologically, particularly the palatopharyngeus, superior constrictor, and levator veli palatini.

**Methods:**

We examined 15 heads from adult Japanese cadavers—12 macroscopically and three histologically.

**Results:**

We observed superior constrictor attaching to the most lateral part of the soft palate and three consistently identifiable fiber groups, classified according to their origin on the superior or inferior surface of the soft palate and their relationship to the levator veli palatini. The superolateral part of the palatopharyngeus originated inferior to the levator veli palatini and ran obliquely and transversely on the pharyngeal wall. The superomedial part of the palatopharyngeus originated from the superior surface of levator veli palatini, ran orthogonally to levator veli palatini, and comprised the palatopharyngeal arch. The inferior part of the palatopharyngeus originated from the inferior surface of the palatine aponeurosis and comprised the palatopharyngeal arch. Moreover, the inferior part of the palatopharyngeus was attached to the base of the epiglottis and thyroid cartilage.

**Conclusions:**

The multidirectional bundles of the palatopharyngeus, superior constrictor, and levator veli palatini within the soft palate are closely related anatomically and play important roles in velopharyngeal closure.

## Introduction

Velopharyngeal closure is caused by elevation of the soft palate and constriction of the pharyngeal isthmus, which blocks the pathway between the nasopharynx and oropharynx.^1^, ^2^, ^3^, ^4^ The levator veli palatini, superior constrictor, and palatopharyngeus play important roles in velar elevation. Among them, the levator veli palatini plays a central role and is considered indispensable for achieving complete velopharyngeal closure.^5^, ^6^, ^7^, ^8^, ^9^, ^10^, ^11^

The cleft lip and palate (CLP) is a birth defect that causes incompetence in velopharyngeal closure. Patients with CLP often present with the following anatomical features: cleft of the soft palate, anteriorly running levator veli palatini, and attachment to the posterior border of the hard palate. Palatoplasty is typically performed to correct these anatomical deficits by closing the cleft, reconstructing the levator veli palatini, and reducing the nasopharyngeal cavity volume. The standard technique involves posterior mobilization, overlapping, and midline suturing of the levator veli palatini to its contralateral counterpart.^12^, ^13^, ^14^ Despite refinements in the surgical technique, postoperative velopharyngeal insufficiency (VPI) persists in approximately 15–25% of patients, and sometimes up to 30% following primary palatoplasty.^15^, ^16^, ^17^, ^18^, ^19^ These findings suggest that additional anatomical considerations may be needed beyond levator veli palatini reconstruction to achieve optimal outcomes. Consequently, secondary interventions, such as the buccal myomucosal flap (BMFF), have been introduced to address persistent VPI.^20^, ^21^, ^22^, ^23^ While the BMFF provides an additional option for managing persistent VPI, attention to potential postoperative complications is warranted, and these risks underscore the need to balance functional reconstruction with the preservation of normal oral and pharyngeal structures.

While the gross anatomy of the pharynx and soft palate has been extensively documented,^24^, ^25^, ^26^, ^27^, ^28^, ^29^, ^30^ the precise spatial relationships among the constituent muscles—particularly the palatopharyngeus, levator veli palatini, and superior constrictor —remain incompletely understood. Although recent studies have applied three-dimensional imaging techniques to map their anatomy,^3^^,^^31^^,^^32^ the lack of distinct anatomical borders between these muscles limits the ability of three-dimensional (3D) reconstructions to resolve their true interrelationships. To clarify such complex anatomical continuity, serial histological sectioning with macroscopic analysis is essential. Thus, further anatomical investigation is warranted to better understand normal velopharyngeal function and postoperative dysfunction. Accordingly, this study aimed to clarify the spatial relationships between the muscle bundles of the soft palate and pharynx macroscopically and histologically, particularly the palatopharyngeus, superior constrictor, and levator veli palatini, and discuss the function of velopharyngeal closure.

## Materials and methods

### Ethics statements

The ethics committee of our institution approved the study design (approval number: D2018-055).

### Cadaver preparation

We evaluated 15 heads from Japanese cadavers (six males and nine females; mean age: 77.5 years, 67–91 year) donated to the Institute of Science Tokyo. Before death, all donors voluntarily donated their remains for educational and research purposes. This study complied with the Japanese law entitled “Act on Body Donation for Medical and Dental Education,” and all procedures were performed in accordance with the “Japanese Ethical Guidelines for Medical and Health Research Involving Human Subjects.”

All cadaver specimens were fixed in 8% formalin and preserved in 30% ethanol. Out of 15 cadaver specimens, 12 were randomly assigned for macroscopic examination and three for histological analysis. None of the cadavers used in this study had a history of craniofacial surgery or any other syndromes.

### Macroscopic examination

Of the 12 heads, seven, two, and three were randomly assigned to posterior, anterosuperior, and anteroinferior dissections, respectively. We classified the palatopharyngeus into three parts based on their origins within the soft palate. We defined the muscle bundle of the palatopharyngeus on the basis of its relationship with the palatine aponeurosis and levator veli palatini as follows: inferior part of the palatopharyngeus, originates from the inferior surface of the palatine aponeurosis; superomedial part of the palatopharyngeus, originates from the superior surface of the palatine aponeurosis, medial to the levator veli palatini; and superolateral part of the palatopharyngeus, originates from the superior surface of the palatine aponeurosis, lateral to the levator veli palatini.

### Dissection from the posterolateral aspect

To observe the origin and course of the superior constrictor entering the soft palate, we removed the lateral plate of the pterygoid process and squamous part of the temporal bone. The muscle bundles in the soft palate and lateral wall of the pharynx were observed.

### Dissection from the posterior aspect

To observe the origins of the superomedial part of the palatopharyngeus, superolateral part of the palatopharyngeus, and superior constrictor in the soft palate, the muscle bundles in the soft palate were dissected posteriorly on the basis of the dissection procedures in a previous study.^3^ The posterior pharyngeal wall was incised along the midline to observe the courses and insertions of the superomedial part of the palatopharyngeus and superolateral part of the palatopharyngeus.

### Dissection from the anteroinferior aspect

To observe the origin and insertion of inferior part of the palatopharyngeus, the anterior pharyngeal wall was also incised on the midline on the basis of dissection procedures in a previous study.^24^ The heads were transected coronally through the nasal cavity at the anterior part of the hard palate, and most of the contents of the maxilla and infratemporal fossa were removed with the tensor veli palatini intact to observe the muscle bundles from the lateral pharyngeal wall to the soft palate.

### Histological examination

To analyze the layered structure of the soft palate, coronal sections of the anterior and inferior levels of the soft palate were stained with Masson trichrome staining. Three halves were used for the histological analysis of the soft palate. We froze specimens at –80°C and serially sectioned them into 5-mm thick segments in the coronal plane using a band saw (WN-25-3; Nakajima Seisakusho, Osaka, Japan). The muscles of the soft palate were harvested en bloc in coronal sections using a diamond saw. The blocks were decalcified for 2 weeks in a solution containing aluminum chloride, hydrochloric acid, and formic acid, as described by Plank and Rychlo. After dehydration, the blocks were embedded in paraffin and serially sectioned at a thickness of 5 µm. Thereafter, the sections were stained with Masson trichrome staining to distinguish collagen fibers from other connective tissues.

## Results

### Macroscopic analysis

#### Superior constrictor entering the soft palate

**Figure 1:**
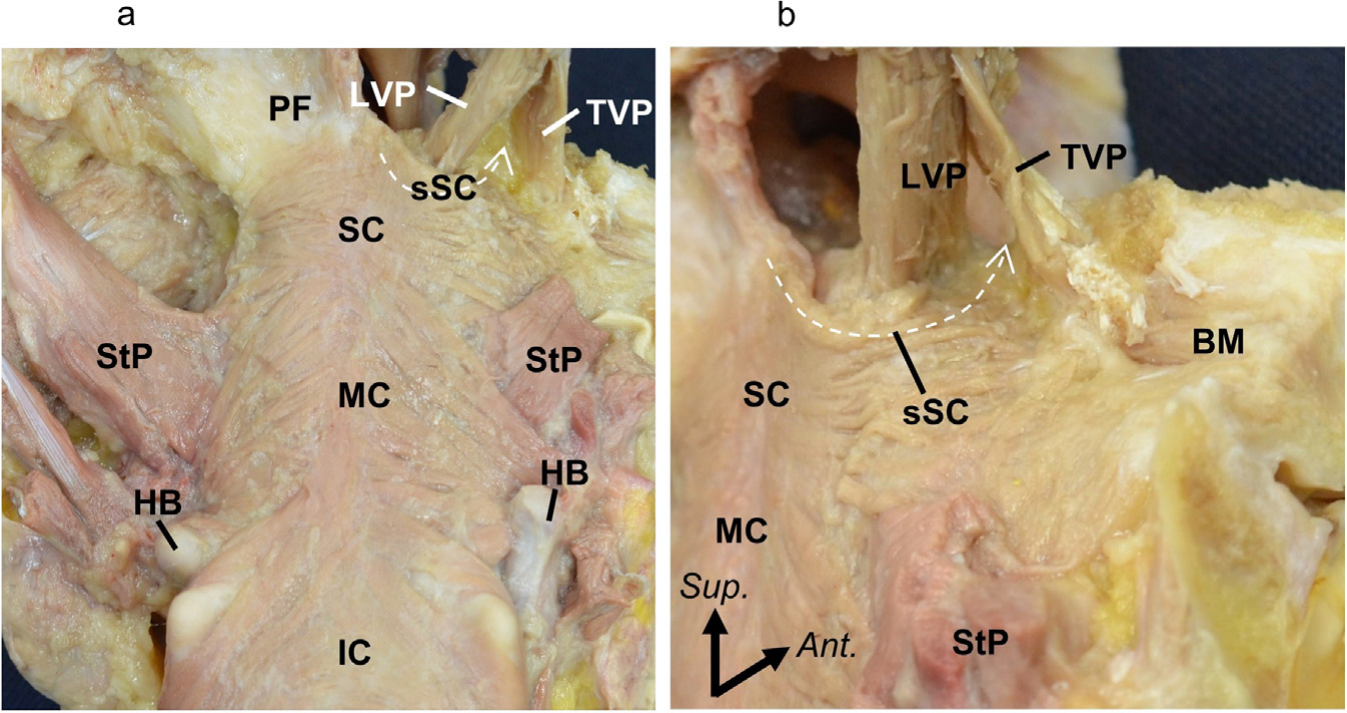
Muscle bundle of the SC entering the soft palate. (a) Posterior surface of the posterior pharyngeal wall. (b) Posterolateral aspect of the superior part of the SC. BM, buccinator; HB, hyoid bone; IC, inferior constrictor; LVP, levator veli palatini; MC, middle constrictor; PF, pharyngobasilar fascia; SC, whole of the superior constrictor; sSC, superior constrictor entering the soft palate; StP, stylopharyngeus; and TVP, tensor veli palatini.

The superior part of the superior constrictor ran and attached to the most lateral part of the soft palate between the tensor veli palatini and levator veli palatini (Figure 1a, b).

#### Palatopharyngeus originating superior to the palatine aponeurosis

**Figure 2:**
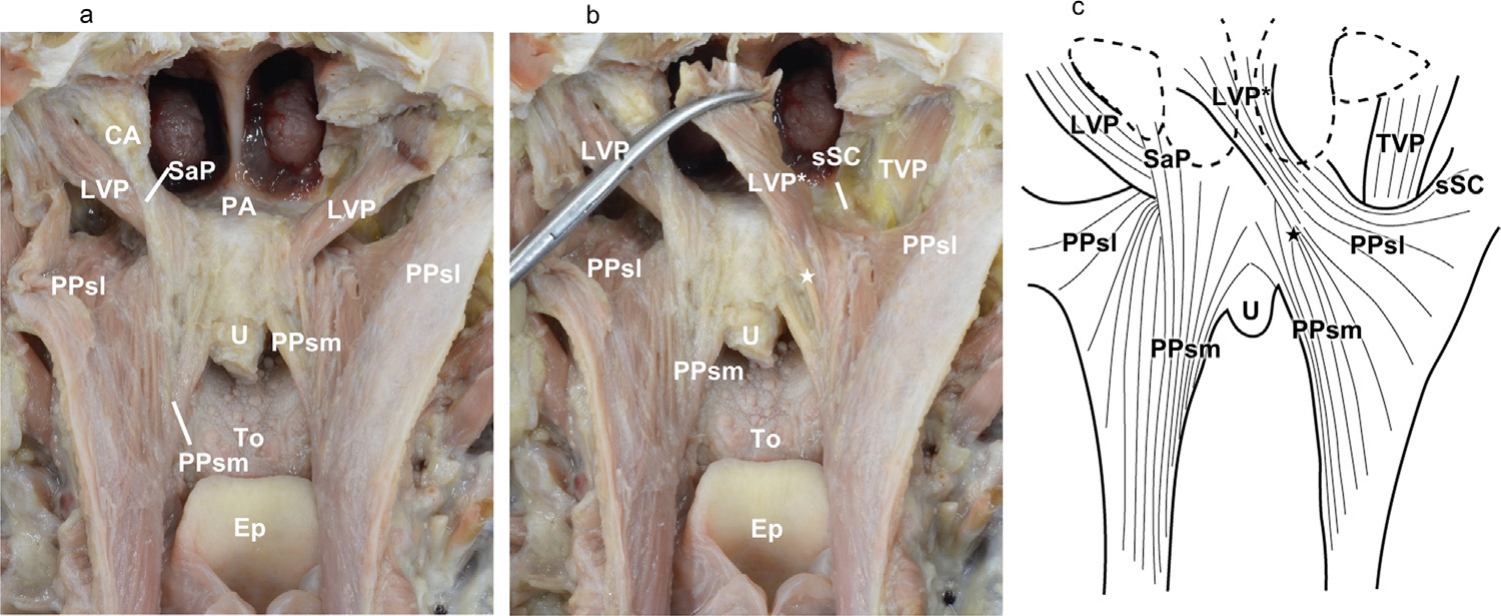
Layered structure of the superior surface of the soft palate (posterior aspect). (a) Posterior aspect of the soft palate and medial surface of the pharyngeal wall after cutting at its midline. (b) Posterior aspect of the soft palate and medial surface of the pharyngeal wall after reflecting the LVP. (c) Schematic representation of Figure 2a and b. CA, cartilage of the auditory tube; Ep, epiglottis; LVP, levator veli palatini; LVP*, levator veli palatini after reflection; PPsl, superolateral part of the palatopharyngeus; PPsm, superomedial part of the palatopharyngeus; SaP, salpingopharyngeus; SC, whole of the superior constrictor; sSC, superior constrictor entering the soft palate; TC, thyroid cartilage; TVP, tensor veli palatini; and U, musculus uvulae. LVP running to the pharyngeal wall is indicated by the white star.

The palatopharyngeus originating superior to the palatine aponeurosis originated from the superomedial and inferolateral surfaces of the levator veli palatini. The superomedial part of the palatopharyngeus originated from the superomedial surface of the levator veli palatini, ran orthogonally to it on the medial part of the soft palate, and extended inferiorly along the inner surface of the pharyngeal wall, forming the posterior part of the palatopharyngeal arch. The superolateral part of the palatopharyngeus was originated from the superior surface of the palatine aponeurosis and inferolateral surface of the levator veli palatini expanded radially on the superolateral part of the inner surface of the pharyngeal wall. The superolateral part of the palatopharyngeus ran parallel to the muscle bundle of the levator veli palatini on the lateral part of the soft palate. Additionally, we observed the salpingopharyngeus from the auditory cartilage to the pharyngeal wall close to the superomedial part of the palatopharyngeus on two of the seven sides (Figure 2a). The attachment site of the superior constrictor entering the soft palate was located anterior to the attachment site of the levator veli palatini. The superolateral part of the palatopharyngeus and superior constrictor entering the soft palate originated from the soft palate between the levator veli palatini and tensor veli palatini, but the border between the superolateral part of the palatopharyngeus and superior constrictor entering the soft palate was unclear on the soft palate, especially in the anterior region. Moreover, the muscle bundle of the levator veli palatini running to the pharyngeal wall was observed (Figure 2b).

#### Palatopharyngeus originating from the inferior surface of the palatine aponeurosis

**Figure 3:**
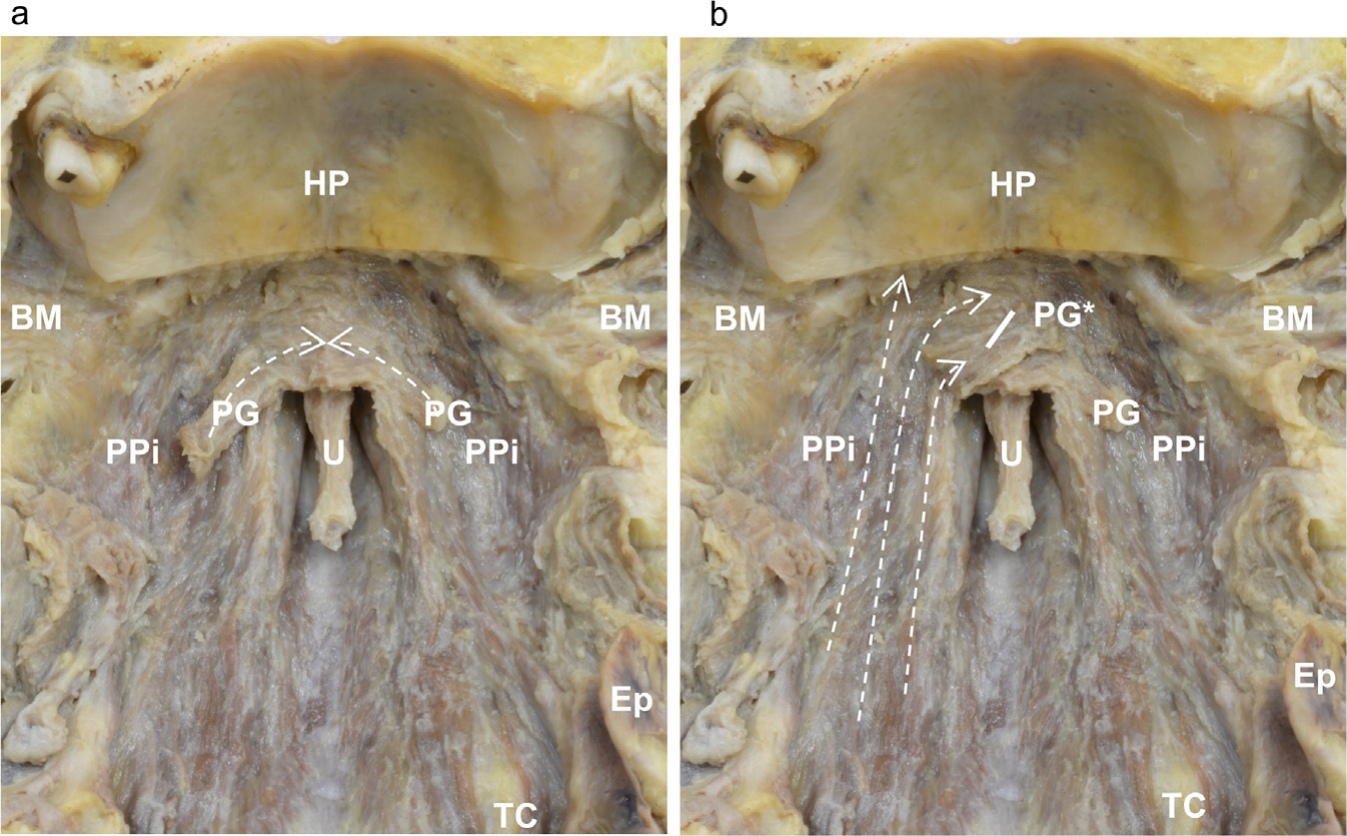
Layered structure of the inferior surface of the soft palate (inferoanterior aspect). (a) Inferoanterior aspect of the soft palate and medial surface of the pharyngeal wall. (b) Inferoanterior aspect of the soft palate and medial surface of the pharyngeal wall after reflecting the palatoglossus. BM, bucinator; Ep, epiglottis; HP, hard palate; PG, palatoglossus; PG*, palatoglossus after reflection; PPi, inferior part of the palatopharyngeus; and U, uvula.

The inferior part of the palatopharyngeus was originated from the inferior surface of the palatine aponeurosis ran posteroinferiorly, formed the anterior part of the palatopharyngeal arch, and attached to the base of the epiglottis and the lateral part of the thyroid cartilage. In the soft palate, the inferior part of the palatopharyngeus entered inferior to the palatoglossus, anterior to the palatoglossus, and adjoined each side of the muscle bundle and palatine aponeurosis (Figure 3a, b).

The three subdivisions of the palatopharyngeus were consistently identified in all 12 specimens, although the clarity and degree of development varied among individuals.

### Histological analysis

**Figure 4:**
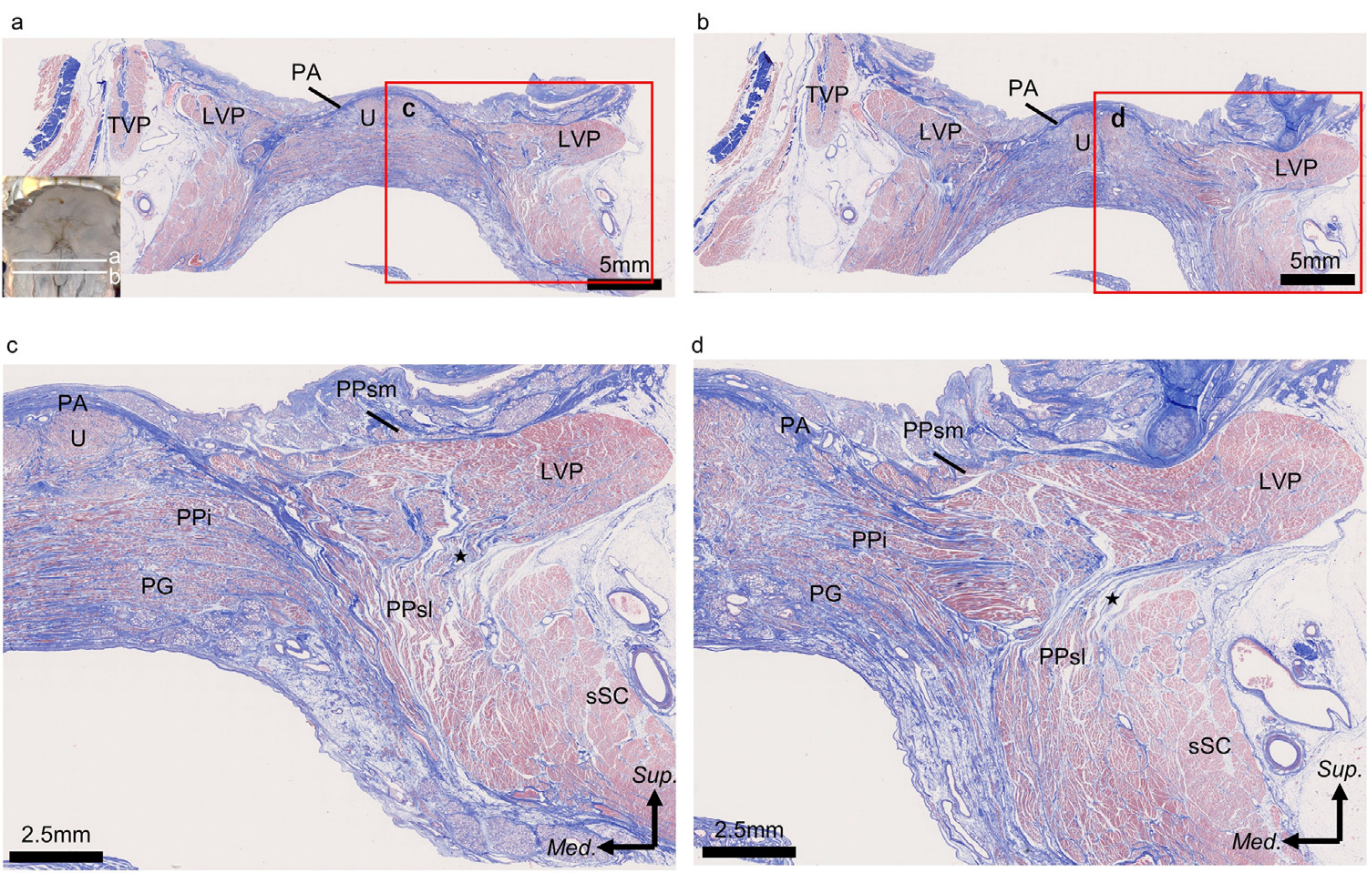
Histological analysis of the layered structure of the soft palate using Masson trichrome staining (coronal section). (a) Coronal section of the soft palate along line a. (b) Coronal section of the soft palate along line b. (c) High magnification image of box C in Figure 4a. (d) High magnification image of box D in Figure 4b. PPi, inferior part of the palatopharyngeus; PPsm, superomedial part of the palatopharyngeus; PPsl, superolateral part of the palatopharyngeus; LVP, levator veli palatini; PA, palatine aponeurosis; sSC, superior constrictor entering the soft palate; TVP, tensor veli palatini; PG, palatoglossus; and U, uvula. *LVP running to the pharyngeal wall is indicated by the black star.

The following structures were observed in the coronally sectioned histological image (posterior soft palate) with Masson trichrome staining. In the midline, the palatine aponeurosis was identified as dense collagen fibers. On the basis of the macroscopic observation, we identified the muscles inferior to the palatine aponeurosis as the uvula, inferior part of the palatopharyngeus, and palatoglossus. Laterally, the levator veli palatini, superior constrictor entering the soft palate, and superolateral part of the palatopharyngeus were observed to be superior to the superior constrictor entering the soft palate and superolateral part of the palatopharyngeus. The border between the superior constrictor entering the soft palate and superolateral part of the palatopharyngeus was unclear (Figure 4a, b). A small slip of the levator veli palatini extended medially to continue into the superolateral part of the palatopharyngeus (Figure 4c, d).

In the medial part of the soft palate, the lateral part of the collagenous fibers of the palatine aponeurosis entered among the muscle bundles of the superolateral part of the palatopharyngeus or levator veli palatini. The inferior part of the palatopharyngeus and palatoglossus, which are located inferiorly to the palatine aponeurosis, were divided by palatine aponeurosis from the levator veli palatini and superolateral part of the palatopharyngeus (Figure 4c). In the lateral part of the soft palate, the collagenous fibers of the palatine aponeurosis entered among the muscle bundles of the inferior part of the palatopharyngeus or levator veli palatini. The muscle bundle of the levator veli palatini interdigitated with the inferior part of the palatopharyngeus and palatoglossus (Figure 4d).

## Discussion

### Main findings

**Figure 5:**
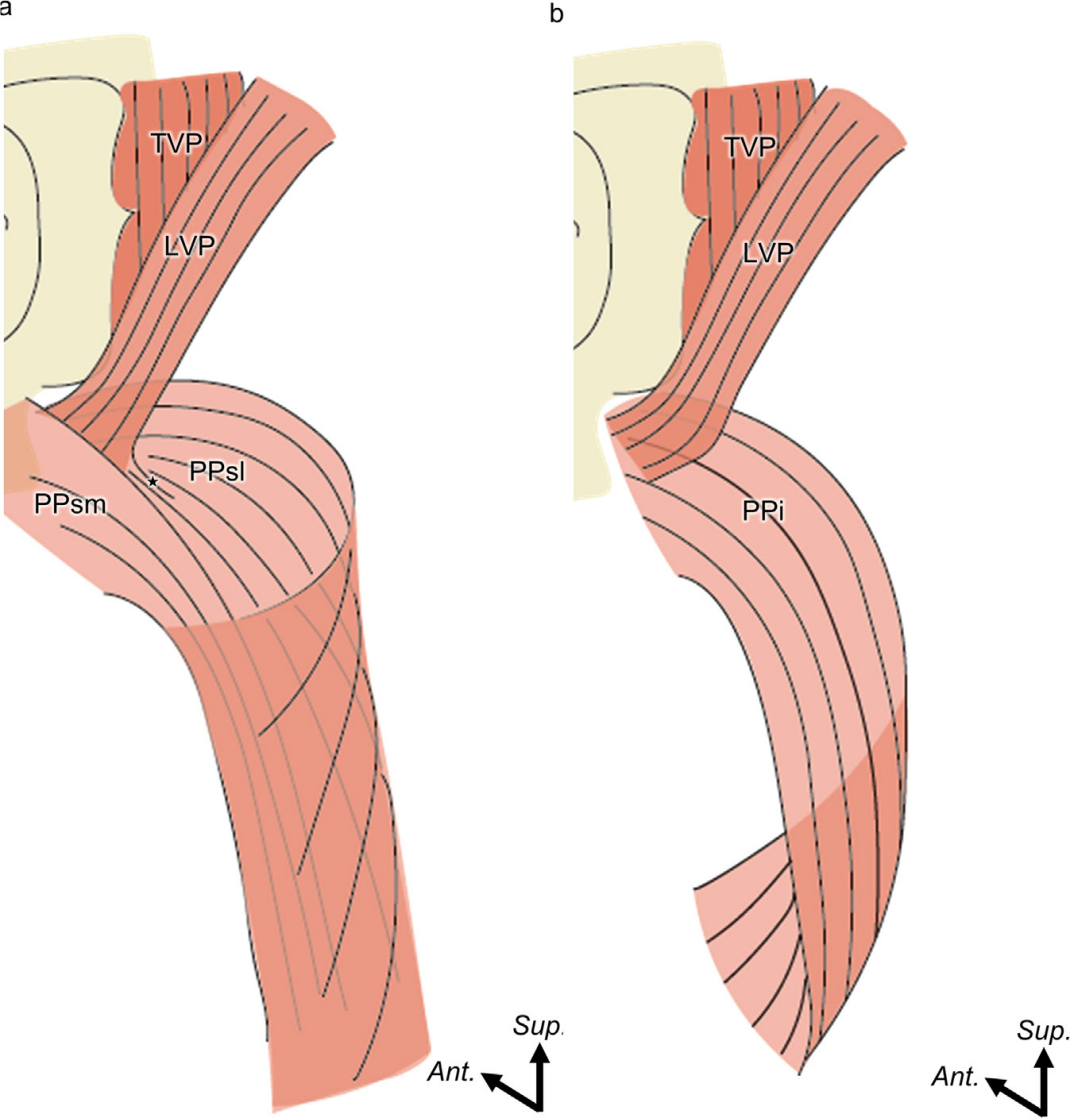
Schematic representation of spatial distribution of the PPsl, PPsm, and PPi (posterolateral aspect). (a) Spatial distribution of the PPi inferior to the PA. (b) Spatial distribution of the PPsl and PPsm superior to the PA. PPi, inferior part of the palatopharyngeus; PPsm, superomedial part of the palatopharyngeus; PPsl, superolateral part of the palatopharyngeus; LVP, levator veli palatini; and TVP, tensor veli palatini.

In the present study, we identified three parts of the palatopharyngeus (superolateral, superomedial and inferior part of the palatopharyngeus) and the superior constrictor entering the soft palate surrounding the levator veli palatini. The muscle fibers of the superolateral part of the palatopharyngeus and inferior part of the palatopharyngeus ran parallel to and were continuous with those of the levator veli palatini. In contrast, the superomedial part of the palatopharyngeus ran orthogonally across the levator veli palatini (Figure 5). These anatomical subdivisions form the basis for understanding their respective contributions to the mechanics of velopharyngeal closure. Although we describe three subdivisions based on origin and fiber orientation, these should be regarded as functional subregions within a continuous muscular complex rather than discrete anatomical compartments.

Previous anatomical studies have proposed various classifications of the palatopharyngeus based on its broad anatomical distribution and complex structure. Okuda et al. categorized the palatopharyngeus by origin and its spatial relation to the levator veli palatini,^24^ whereas Hwang et al. emphasized its topography around the levator veli palatini.^25^ Cho et al. proposed a functional classification based on fiber orientation,^26^ and Sakamoto et al. refined their classification by analyzing 3D trajectories and the positional depth of the fiber bundles.^27^ While these classifications have supported our anatomical understanding, they largely conceptualize the palatopharyngeus as a sum of distinct units. In contrast, our findings demonstrate that the palatopharyngeus muscle bundles are continuous, multidirectional, and tightly interwoven without distinct histological boundaries. This supports the idea that the palatopharyngeus should be understood not as a set of separate compartments, but as a single functionally integrated muscular complex. Such reinterpretation may offer a more coherent anatomical basis for understanding the biomechanics of velopharyngeal closure.

### Mechanism of velopharyngeal closure

**Figure 6:**
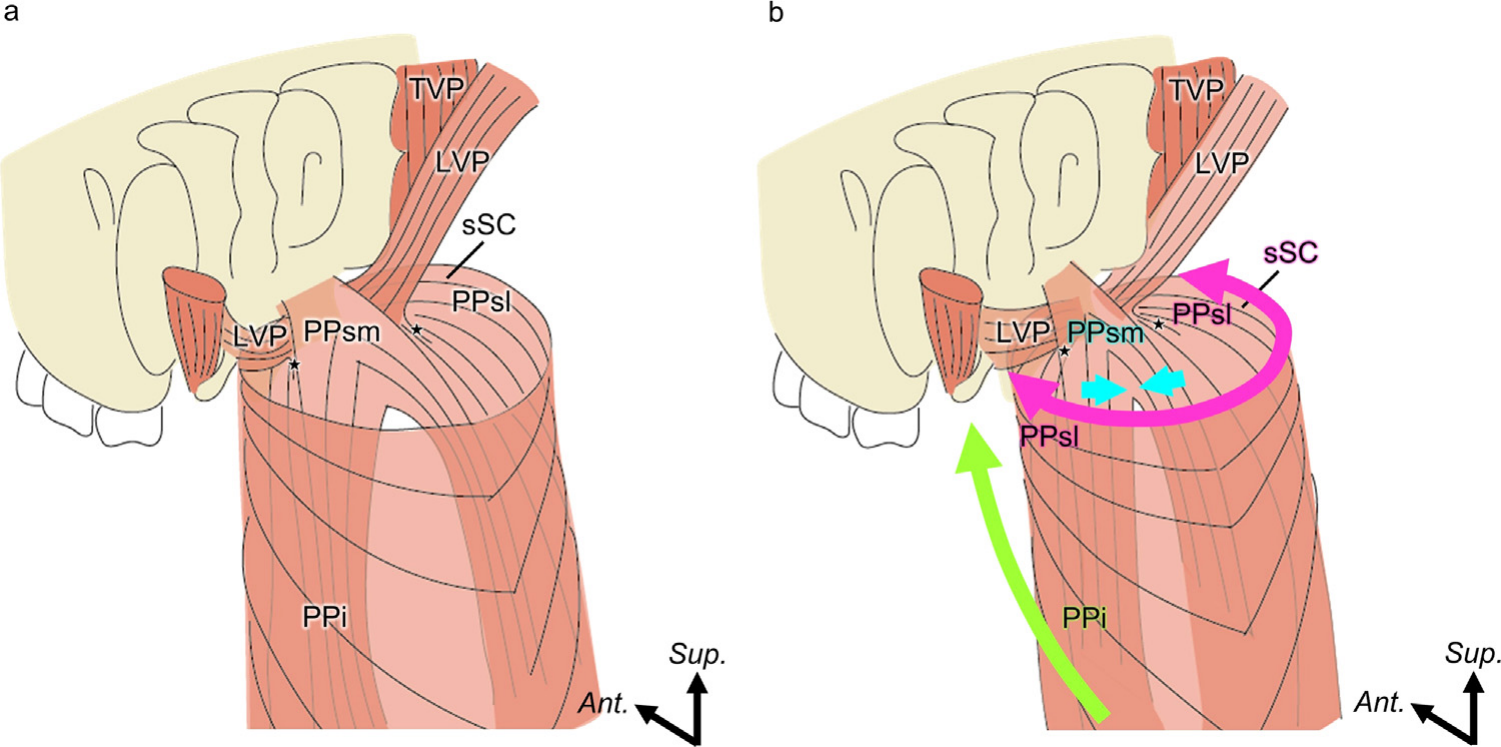
Schematic representation of the muscles of the soft palate and velopharyngeal closure (posterolateral aspect). (a) At rest. (b) At velopharyngeal closure. The pink arrows indicate constriction of the pharyngeal isthmus by the sSC and PPsl. The blue arrows indicate the medial protrusion of the palatopharyngeal arch by the PPsm. The green arrows indicate shortening of the pharynx caused by the PPi. PPi, inferior part of the palatopharyngeus; PPsm, superomedial part of the palatopharyngeus; PPsl, superolateral part of the palatopharyngeus; LVP, levator veli palatini; and TVP, tensor veli palatini; sSC, superior constrictor entering the soft palate.

Velopharyngeal closure involves elevation of the soft palate, constriction of the pharyngeal isthmus—often attributed primarily to the levator veli palatini—and medial protrusion of the pharyngeal walls.^1^, ^2^, ^3^^,^^33^ This process is not driven by any single muscle but is achieved through the integrated and simultaneous actions of the palatopharyngeus, levator veli palatini, and superior constrictor entering the soft palate (Figure 6).

On the basis of fiber orientations and attachment sites, we propose differentiated roles for each subdivision of the palatopharyngeus. The superomedial part of the palatopharyngeus, which crosses over the levator veli palatini, may contribute to medial displacement of the palatopharyngeal arch. The superolateral part of the palatopharyngeus, which runs parallel to the levator veli palatini, may coordinate with the superior constrictor entering the soft palate to constrict the pharyngeal isthmus. The inferior part of the palatopharyngeus, which inserts into the epiglottis and thyroid cartilage, may assist in pharyngeal narrowing and vertical laryngeal elevation. Acting in concert with the surrounding muscles, these subdivisions dynamically reshape the soft palate and pharyngeal wall to achieve effective velopharyngeal closure. This multidirectional arrangement shifts the perspective from viewing the palatopharyngeus as a simple longitudinal elevator to recognizing it as a structurally layered and functionally multifaceted muscle.

### Clinical implications and surgical considerations

The close anatomical and spatial relationship among the palatopharyngeus, levator veli palatini, and superior constrictor entering the soft palate underscores the need to consider these muscles collectively, particularly in surgical planning for patients with CLP. In patients with CLP, the levator veli palatini runs more anteriorly and laterally, and that the muscular sling fails to form a continuous arch across the midline but instead attaches abnormally to the posterior margin of the hard palate.^31^^,^^32^, ^34^, ^35^, ^36^ Histological and morphometric studies have further shown hypoplasia of the palatine aponeurosis and altered muscle fiber composition compared with the intact palate.^37^^,^^38^ Together, these findings indicate that the cleft palate possesses a distinct anatomical and physiological organization, not a simple variant of normal morphology. Whether the characteristic layered muscle architecture of the soft palate in this study is preserved in patients with CLP remains uncertain, we could demonstrate that the levator veli palatini, superior constrictor entering the soft palate, and palatopharyngeus are not only closely positioned but also intermingled at least in normal anatomy, presenting potential challenges for surgical identification and preservation. While levator veli palatini-focused reconstruction has markedly improved speech outcomes and remains central to cleft palate repair, residual VPI still occurs in a subset of patients. This suggests that the palatopharyngeus and superior constrictor entering the soft palate may also play important roles in achieving stable velopharyngeal closure. Rather than altering current techniques, incorporating a deeper understanding of these muscular interrelations, particularly their layered architecture, may help refine surgical approaches and improve outcomes in persistent VPI cases.

### Limitations

This study has a few limitations. The earlier part of the study was an anatomical investigation using cadaveric specimens; thus, age related changes in the muscle physiology could not be assessed. Because the specimens were elderly (mean age 77.5 years), muscle elasticity and thickness may differ from those in younger individuals; however, previous anatomical and MRI studies have indicated that the basic arrangement and layered structure of the soft palate muscles remain largely unchanged with age.^39^^,^^40^ Therefore, this study focused on these structural configurations rather than tissue elasticity.

In addition, future studies involving pediatric and CLP patients, as well as in vivo MRI and endoscopic evaluations, are planned to clarify the functional dynamics and to explore potential applications for surgical reconstruction.

### Conclusions

This study revealed that within the soft palate, the superomedial part of the palatopharyngeus runs orthogonally on the superior surface of the levator veli palatini, while the superolateral part of the palatopharyngeus and inferior part of the palatopharyngeus extend toward the levator veli palatini. The close anatomical relationships and multidirectional bundles of the palatopharyngeus, superior constrictor, and levator veli palatini within the soft palate and pharyngeal wall play essential roles in velopharyngeal closure. This deeper understanding of soft palate muscle architecture has direct implications for improving surgical outcomes and refining interventions for velopharyngeal dysfunction.

## Author contributions

Fukino K: Conceptualization, study design, data interpretation, manuscript writing, and supervision. Tsutsumi M: Methodology, cadaveric dissection, data acquisition, and initial draft preparation. Kinoshita Y: Data collection and analysis support. Matsumoto Y: Critical revision of the manuscript. Ono T: Supervision and critical review of the final manuscript. Iwanaga J: Supervision, data interpretation, and critical review of the final manuscript. Akita K: Project supervision, anatomical expertise, and final approval of the manuscript.

## Funding

None.

## Ethical approval

The Ethics Committee of the 10.13039/501100010795Institute of Science Tokyo approved the study design (approval number: D2018-055).

## Data availability

All data generated or analyzed during this study are included in this published article.

## Declaration of competing interest

None declared.
